# Performance Characteristics and 2-Year Outcomes of Digital Tomosynthesis-Assisted Electromagnetic Navigation Bronchoscopy in a Community Hospital

**DOI:** 10.1016/j.chpulm.2026.100269

**Published:** 2026-05-25

**Authors:** Elizabeth M. Sain, Brandon T. Rapier, Michael J. Forte, Anuja L. Sarode, Amber E. Kerstetter-Fogle, Valerie Slezak, Brian D. Bauman

**Affiliations:** a Summa Health System Pulmonary Disease and Critical Care Medicine Fellowship Program, Akron, OH; b Summa Health System Internal Medicine Residency Program, Akron, OH; c Pulmonary and Critical Care Medicine, Summa Health System Pulmonary Disease and Critical Care Medicine Fellowship Program, Department of Pulmonary and Critical Care Medicine, Summa Health System, Akron, OH; d Summa Health, Research and Innovation, DISCOVER Center, Akron, OH; e Summa Health System, Akron, OH; f Pulmonary and Critical Care Medicine, Pulmonary Service Line, Respiratory Care, and Pulmonary Nodule Program, Summa Health System, Akron, OH

**Keywords:** diagnostic accuracy, diagnostic yield, digital tomosynthesis-assisted ENB, DT-ENB, electromagnetic navigation bronchoscopy, ENB, lung nodule, lung nodule biopsy

## Abstract

**Background:**

The long-term performance characteristics of digital tomosynthesis-assisted electromagnetic navigation bronchoscopy (DT-ENB) has not been evaluated in the community setting.

**Research Question:**

What is the diagnostic yield and 2-year diagnostic accuracy of DT-ENB in a typical pulmonary practice?

**Study Design and Methods:**

This was a single-center, community hospital-based retrospective observational performance evaluation over 2 years assessing the long-term diagnostic accuracy of DT-ENB using the ILLUMISITE Fluoroscopic Navigation Platform (Medtronic). Other performance characteristics assessed included diagnostic yield, negative predictive value (NPV), and sensitivity. Factors affecting diagnostic yield were analyzed, and subsequent workup for false negatives and outcomes were described.

**Results:**

A total of 227 patients underwent DT-ENB, resulting in a specific diagnosis for 175 patients, for a diagnostic yield of 77.1% (95% CI, 71.2-82.1). Of these, 202 patients either had a confirmatory diagnosis or 2 years of CT scan follow-up. Among the 202 patients included in the NPV and sensitivity analysis, 135 (66.8%) were determined to be true positives, 47 (23.3%) were true negatives, 20 (9.9%) were false negatives, and there were no false positives. Overall, 2-year diagnostic accuracy was 71.4% (95% CI, 65.2-76.9). The 2-year NPV was 70.1% (95% CI, 61.0-78.0), and sensitivity was 87.1% (95% CI, 80.8-91.9). Nodule size was associated with higher diagnostic yield (*P* = .001), with a median nodule size for nondiagnostic procedures of 1.5 cm (95% CI, 1.25-2.15 cm) and a median nodule size for diagnostic procedures of 2.2 cm (95% CI, 1.4-4.0 cm).

**Interpretation:**

Our results show that DT-ENB when used in the community setting for lung nodule biopsy resulted in a diagnostic yield of 77.1% and diagnostic accuracy of 71.4% at 2-year follow-up.

**Take-Home Points**

**Study Question:** In the community setting, what is the diagnostic yield and what is the 2-year diagnostic accuracy, sensitivity, and negative predictive value of digital tomosynthesis-assisted electromagnetic navigation bronchoscopy?

**Results:** The diagnostic yield was 77.1% by the strict definition. The 2-year diagnostic accuracy using the conservative definition was 71.4%, and sensitivity and negative predictive value for malignancy were 87.1% and 70.1%, respectively.

**Interpretation:** In this retrospective observational performance evaluation, digital tomosynthesis-assisted electromagnetic navigation bronchoscopy performed well in the community setting, with 2-year data consistent with other benchmark studies, despite using conservative definitions for diagnostic yield and accuracy.

In recent years, utilization of electromagnetic navigation bronchoscopy (ENB) and robotic bronchoscopy has largely superseded other diagnostic modalities for the assessment of lung nodules, given its favorable diagnostic yields and excellent safety profile.^1^, ^2^, ^3^, ^4^ Multiple large multicenter studies have examined the yield of ENB. The Clinical Evaluation of superDimension™ Navigation System for Electromagnetic Navigation Bronchoscopy (NAVIGATE) study demonstrated a yield of 67.8% and a sensitivity of 64.7% using the superDimension Navigation System (Medtronic).^5^ Another large meta-analysis, with most studies using the superDimension Navigation System, demonstrated the overall sensitivity of ENB at 77%.^6^ The Navigation Endoscopy to Reach Indeterminate Lung Nodules Versus Trans-Thoracic Needle Aspiration (VERITAS) trial compared the yield of ENB using the ILLUMISITE Fluoroscopic Navigation Platform (Medtronic) with CT scan-guided transthoracic needle biopsy.^7^ The accuracy of the ENB group, defined as the percentage of specific benign or malignant pathologic diagnoses confirmed at 12 months, was found to be 79% vs 73.6% in the CT scan-guided group.^7^ These trials reported a maximum follow-up of 1 year.^5^, ^6^, ^7^

Assessing yield across various data sets, follow-up lengths, and trials is challenging. The American Thoracic Society (ATS)/American College of Chest Physicians (CHEST)^8^ released new, stricter guidelines to better standardize reporting, which may have impacted some prior reported yields. In addition, prolonged follow-up periods up to 2 years have not been studied.

Digital tomosynthesis-assisted electromagnetic navigational bronchoscopy (DT-ENB) integrates fluoroscopic technology to accurately visualize a target location and increase yield.^9^ Historically, CT-to-body divergence, or the mismatch between nodule location on preprocedural CT scan and its location during the procedure, limited diagnostic yield.^9^^,^^10^ Factors that impact CT-to-body divergence include differences in lung inflation under positive pressure ventilation, tissue distortion related to the bronchoscope, and lung excursion during respiration.^10^, ^11^, ^12^ CT-to-body divergence is greater for nodules nearer to the diaphragm more affected by atelectasis and lung excursion.^10^, ^11^, ^12^ An additional barrier with bronchoscopic biopsies has been the lack of continuous visual guidance via imaging during the procedure.^1^ This results in inaccurate lesion localization and decreased diagnostic yield.^11^

DT-ENB mitigates these barriers with advanced visualization and continuous real-time navigational guidance during the procedure.^9^ Before the procedure, an updated CT scan is obtained. The CT data are then loaded and overlapped with the patient’s bronchial anatomy in real time.^11^ Once the lesion is located, local registration is launched.^11^ This uses fluoroscopy over the lung parenchyma, which are captured via 50° C-arm rotation in conjunction with a proprietary algorithm.^10^^,^^12^ This system allows visualization of the lesion in real time and updates the target intraprocedurally which allows the operator to adjust the catheter for better access and accuracy.^12^^,^^13^ Digital tomosynthesis takes multiple single place radiographic images over a smaller angle (as small as 50°) vs conventional CT scans, which obtain images at 180° to 360°.^14^^,^^15^ The images are obtained in a single sweep of the C-arm and then reconstructed into cross-sectional slices.^15^ Visualization with digital tomosynthesis is improved compared with conventional CT scan.^14^, ^15^, ^16^ This is because of the removal of artifacts from substances such as bone or metal and reduced tissue overlap.^14^, ^15^, ^16^ The amount of radiation from tomosynthesis is a fraction compared with CT scan.^6^^,^^9^ Tomosynthesis typically provides 3-dimensional imaging at an effective radiation dose only approximately double that of a standard chest radiograph.^17^, ^18^, ^19^

Prior studies using DT-ENB have reported yields ranging from 67% to 87%, but they have used variable definitions of diagnostic yield.^6^^,^^9^^,^^20^, ^21^, ^22^, ^23^ To our knowledge, this electromagnetic navigation platform has not been evaluated in a community setting with reported data on 2 years of longitudinal follow-up for each patient. We present the diagnostic yield, diagnostic accuracy, sensitivity, and negative predictive value (NPV) in 227 patients at a single-center community hospital with 2 years of follow-up.

## Study Design and Methods

This study was a single-center, retrospective observational performance evaluation. A total of 227 patients underwent DT-ENB at Summa Health, a large community hospital in Akron, Ohio. This study was approved by the Summa Health System institutional review board (No. 23276). The ILLUMISITE Fluoroscopic Navigation Platform (version 1.0) was used. Patients were included in the study if DT-ENB was performed for lung nodule biopsy; patients who underwent biopsy with endobronchial ultrasound (EBUS) alone were excluded. Procedures were performed by 4 attending physicians with 1 to 10 years of postfellowship bronchoscopy experience (approximately 1, 3, 5, and 10 years, respectively). Radial EBUS probe was not used. Rapid on-site cytology was used for all cases. Demographic data, PET scan avidity, presence of adenopathy, nodule size and location, diagnosis, and follow-up CT results were obtained for all patients treated between September 2020 and April 2022. Standard uptake value (≥ 2.5) was considered PET scan positive. Adenopathy was defined as intrathoracic lymph node ≥ 1.0 cm. Table 1 shows relevant patient and nodule characteristics, including nodule size, location, morphology (ground glass, solid, cavitary, or mixed), and whether spiculation was present. Patient history of smoking and previous cancer were also noted.

**Table 1:** Demographic Characteristics (N = 227)

Characteristic	Value
Age, y	67.9 [10.2]
Sex	
Female	124 (54.62)
Male	103 (45.37)
History of smoking	
Yes	192 (84.58)
No	35 (15.42)
History of cancer	
Yes	66 (29.07)
No	161 (70.93)
Spiculated	
Yes	118 (51.98)
No	109 (48.02)
Nodule size, cm	
< 1	14 (6.17)
1-3	140 (61.67)
3-5	40 (17.62)
5-7	19 (8.37)
> 7	14 (6.17)
Location	
Right upper lobe	72 (31.72)
Right lower lobe (RLL)	52 (22.91)
Right middle lobe (RML)	12 (5.29)
Left upper lobe	56 (24.67)
Left lower lobe	33 (14.54)
Bilateral (B/L) (n = 1) and RML/RLL (n = 1)	2 (0.88)
Nodule type	
Solid	190 (83.70)
Cavitary	9 (3.96)
Ground glass	8 (3.52)
Mixed cavitary/solid	11 (4.85)
Mixed solid/ground glass	9 (3.96)
PET scan positivity (SUV > 2.5)	
Yes	138 (60.79)
N	15 (6.61)
NA	74 (32.60)

Data are presented as mean [SD] or No. (%). SUV = standard uptake value.

CT scans were uploaded by disk to the ILLUMISITE software before the procedure. A pathway through the airways to the lung nodule or mass was planned using the proprietary software. Patients were endotracheally intubated and placed under general anesthesia, and electromagnetic sensors were placed over the patient’s thorax. An initial airway inspection was completed with bronchoscopy. After recruitment maneuvers with 3 30-second breath holds at tidal inspiratory volume (20-mm Hg positive pressure), an ENB catheter with a locatable guide was placed into the working channel of the bronchoscopy to complete local registration of airway landmarks. An ENB catheter was then advanced within 2 cm of the target lesion, at which point a 50° C-arm rotation with fluoroscopy, or sweep, was performed. The target lesion was marked in the software interface based on its visually identified location on fluoroscopy, allowing for updated targeting on the proprietary software. An ENB catheter was advanced to the updated target lesion, at which point the locatable guide was removed, and biopsy tools were placed through the ENB catheter. Nodules were subsequently biopsied with multiple passes using a combination of transbronchial needle aspiration (TBNA), transbronchial forceps biopsies, brushings, and bronchoalveolar lavage with rapid on-site pathology to review initial TBNA and forceps biopsies. An EBUS with lymph node TBNA was subsequently performed for lung cancer staging purposes.

Diagnostic yield was defined as the number of patients in whom the ENB result established a specific malignant or benign diagnosis that guided a concrete treatment plan divided by the total number of patients undergoing biopsy as per 2023 ATS/CHEST recommendations (joint ATS/CHEST guidelines).^8^ Pathology results were categorized as malignant, benign, or nondiagnostic. Malignant results were defined as any result yielding a conclusive diagnosis of cancer. Benign results were defined as any nonmalignant but discrete finding yielding an immediate diagnosis and treatment plan, and included granulomas and infectious etiologies. Nondiagnostic results included atypical cells not clearly diagnostic of malignancy, nonspecific acute and chronic inflammation, normal lung tissue, fibrosis, and necrosis.

Patients with benign and nondiagnostic results were followed in the lung nodule clinic with clinical and radiographic follow-up over a 2-year period. These data were used to calculate 2-year diagnostic accuracy, sensitivity, and NPV for malignancy. For sensitivity and NPV calculations, patients were categorized into true positives, false negatives, and true negatives. Biopsies initially diagnostic for malignancy were considered true positives; those nondiagnostic and subsequently determined malignant on surgical, CT scan-guided transthoracic biopsy, or repeat ENB were considered false negatives; and those which were smaller or unchanged on 24-month follow-up CT scan and thus deemed to be benign were considered true negatives. Patients who were lost to follow-up or died before a conclusive diagnosis were included in the calculation of diagnostic accuracy. Diagnostic accuracy was calculated as the number of patients whose initial diagnosis was confirmed to be accurate at 2 years, divided by the total number of patients who completed initial biopsy. Initial pathology results, subsequent workup, and final diagnosis were queried for nodules with initially nondiagnostic results that were eventually determined to be malignant. New nodules identified or biopsied after follow-up imaging were not included in the analyses.

**Figure 1:**
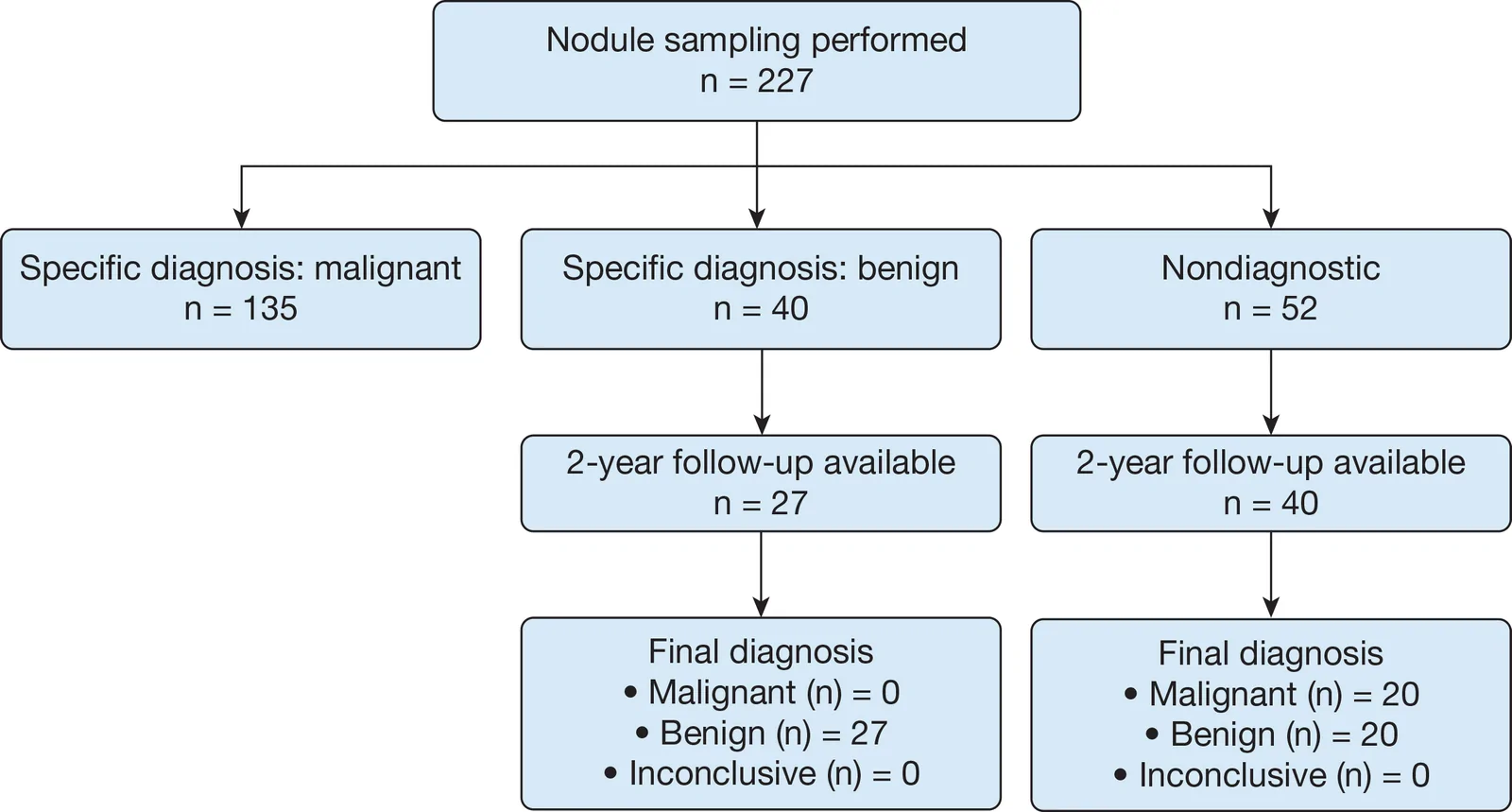
Follow-up pathway after digital tomosynthesis-assisted electromagnetic navigational bronchoscopy.

The follow-up pathway after initial DT-ENB and ultimate outcomes are summarized in Figure 1. We calculated NPV, positive predictive value, sensitivity, specificity,^14^ and diagnostic accuracy using standard formulae.^24^^,^^25^

**Table 9:** Equations Used for Performance Characteristics

Performance Characteristics Equations
Negative predictive value = true negatives/(true negatives + false negatives)
Positive predictive value = true positives/(true positives + false positives)
Sensitivity = true positives/(true positives + false negatives)
Specificity = true negatives/(true negatives + false positives)
Diagnostic yield (at index procedure) = malignant + specific benign pathology/total number of biopsies

**Table 10:** Equation for Diagnostic Accuracy

Diagnostic Accuracy Equation
Diagnostic accuracy (conservative definition) = (true positives + true negatives^a^ at 2-y follow-up/total number of biopsies)

a True negatives are defined as having a specific benign pathology result on index bronchoscopy and documented nodule stability at 2 y.

To compare distributions between nondiagnostic and diagnostic yield groups, the Wilcoxon rank sum test was used. To compare categorical variables between groups (PET scan > 2.5 and location), the χ^2^ test of association was used. *P* < .05 was considered statistically significant (Table 4). CIs for diagnostic yield and diagnostic accuracy were calculated using binomial proportion methods (Wilson score). Descriptive statistics are provided as absolute frequencies and percentages in Tables 5 and 6. All statistical analyses were performed using SAS 9.4.

**Figure 2:**
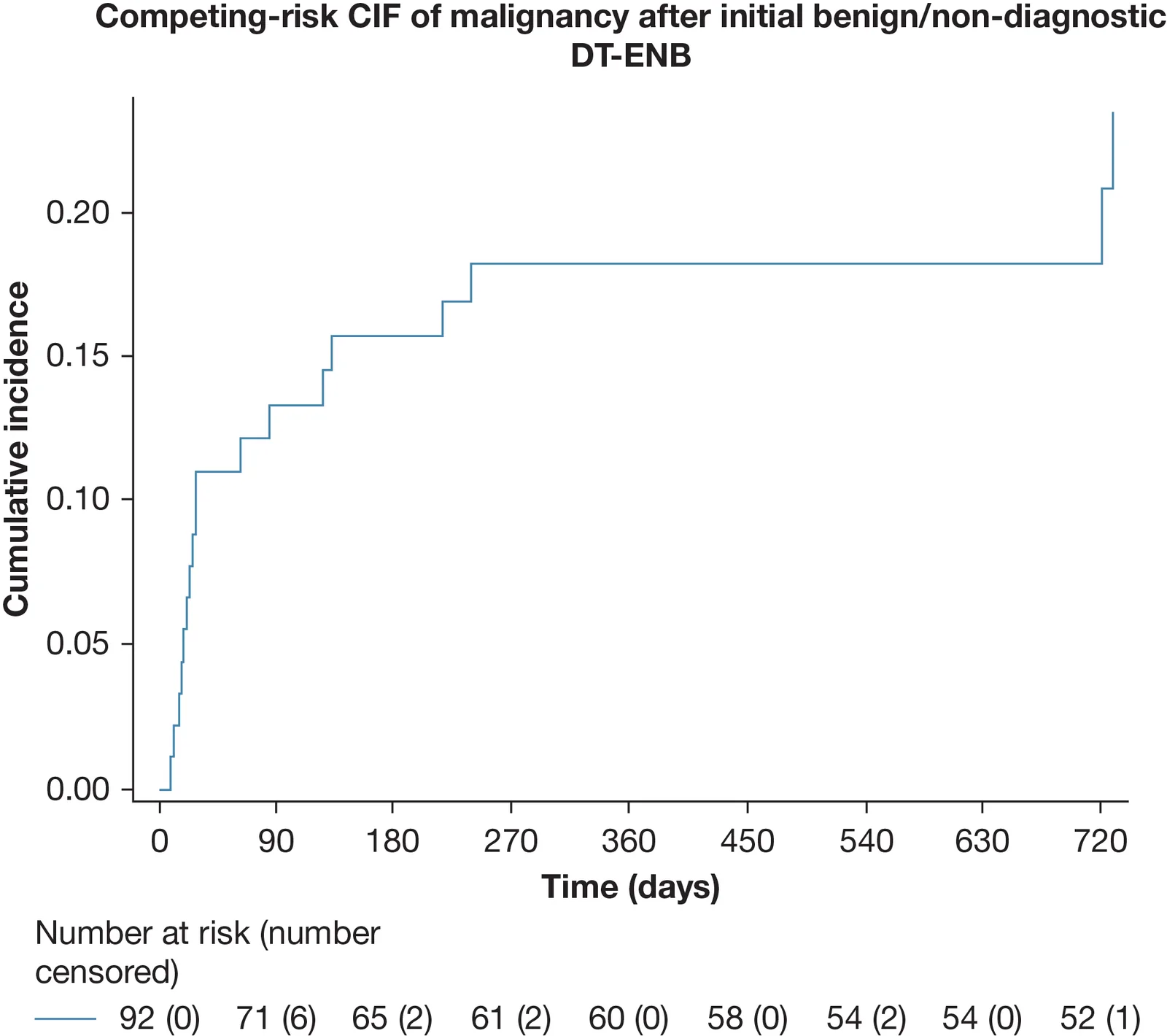
Competing-risk cumulative incidence of malignancy after initial benign/nondiagnostic DT-ENB. CIF = cumulative incidence function; DT-ENB = digital tomosynthesis-assisted electromagnetic navigational bronchoscopy

To visualize diagnostic performance over the follow-up period, we conducted a time-to-event analysis focused on subsequent malignancy confirmation after an initial nonmalignant DT-ENB result (Fig 2). The analysis was restricted to patients whose index DT-ENB biopsy result was benign or nondiagnostic/inconclusive. Time zero was defined as the date of the index DT-ENB procedure. Follow-up time was calculated from the index procedure date to the earliest of (1) date of malignancy confirmation (event of interest), (2) date of death (competing event), (3) date of last known follow-up for those lost to follow-up, or (4) administrative end of follow-up at 2 years. Patients who had a final benign determination, were lost to follow-up, or had no malignancy confirmation by 2 years were treated as censored at the corresponding date. Because death precludes subsequent malignancy confirmation, death was modeled as a competing risk, and we estimated the cumulative incidence function (CIF) for malignancy over time. The CIF curve is presented with the number at risks (and number censored) at prespecified time points. Analyses were performed in SAS (PROC LIFETEST). The CIF can be interpreted as the cumulative proportion of initially nonmalignant DT-ENB cases subsequently confirmed malignant over follow-up, accounting for the competing risk of death.

**Table:** 

Total (No.)	Failed Events (Malignancy)	Competing Events (Death)	Censored
92	20	12	60

Because it is impossible to determine what the final diagnosis would have been for patients who died or were lost to follow-up, and the effect on diagnostic accuracy, NPV, and sensitivity, both best case (all nodules benign) and worst case (all nodules malignant) scenarios were calculated.

## Results

There were 227 patients included in the sample for calculation of strict diagnostic yield. A total of 135 biopsies (59.5%) resulted in diagnosis of malignancy, 40 biopsies (17.6%) resulted in diagnosis of benign etiologies, and 52 biopsies (22.9%) were nondiagnostic. A total of 175 samples were diagnostic, resulting in a diagnostic yield of 77.1% (95% CI, 71.2-82.1) (Table 2).

**Table 2:** Diagnostic Performance Metrics and Initial Pathologic Outcomes for Malignancy Assessment

Initial Outcome	No.
Malignant	135
Benign	40
Nondiagnostic	52
Total	227
Diagnostic yield (strict) (95% CI)	77.1 (71.2-82.1)


Performance Characteristics for Malignancy Diagnosis	Point Estimate (95% CI)
Negative predictive value	70.2 (61.0-78.0)
Positive predictive value	100.0 (97.3-100.0)
Sensitivity	87.1 (80.8-91.9)
Specificity	100 (92.5-100.0)

There were 202 patients who either had a confirmatory diagnosis or 2 years of CT scan follow-up and were included in diagnostic accuracy and NPV analysis. Of these 202 patients, 135 (66.8%) were determined to be true positives, 47 (23.3%) were true negatives, 20 (9.9%) were false negatives, and no false positives were identified in this sample (Table 3). Two-year diagnostic accuracy was 71.4% (95% CI, 65.2-76.9), with an NPV of 70.1% (95% CI, 61.0-78.0) and a sensitivity of 87.1% (95% CI, 80.8-91.9). A total of 25 patients were either lost to follow-up or died before a conclusive diagnosis at 2 years. Nodule size was associated with higher diagnostic yield (*P* = .001), with a median nodule size for nondiagnostic procedures of 1.5 cm (95% CI, 1.25-2.15 cm) and a median nodule size for diagnostic procedures of 2.2 cm (95% CI, 1.4-4.0 cm). PET scan positivity and nodule location were not statistically significant (Table 4).

**Table 3:** Two-Year Follow-Up by Result Category

**Malignant**		**Benign**	
Malignant	135		
**Nondiagnostic**			
Malignant	20	Malignant	0
Benign	20	Benign	27
Lost to follow-up	7	Lost to follow-up	6
Died	5	Died	7
**Diagnostic accuracy (95% CI)**	**71.4 (65.2-76.9)**

**Table 4:** Comparison of Nodule Characteristics and Location by Diagnostic Yield

Nodule Characteristic	Nondiagnostic	Diagnostic	*P* Value
	(n = 52)	(n = 175)	
Nodule size (cm)	1.50 (1.25-2.15)	2.2 (1.4-4.0)	.001
PET scan > 2.5	26 (50.00)	112 (64.37)	.062
Location			
Left lower lobe	7 (13.46)	26 (14.94)	.115
Left upper lobe	17 (32.69)	39 (22.41)	
Right lower lobe	13 (25.00)	39 (22.41)	
Right middle lobe	5 (9.62)	8 (4.60)	
Right upper lobe	10 (19.23)	62 (35.63)	

Data are presented as No. (%), median (interquartile range), or as otherwise indicated.

Benign results on initial pathology (Table 5) included granulomas (25%), bacterial infection (38%), fungal infection (13%), mycobacterial infection (5%), organizing pneumonia (15%), radiation fibrosis (3%), and lymphoid interstitial pneumonia (3%). Nondiagnostic findings (Table 6) included atypical cells (n = 25; 48.1%), normal lung/airway (n = 16; 30.8%), inflammation (n = 7; 13.5%), fibrosis (n = 3; 5.8%), and necrosis (n = 1; 1.9%). One-half of all nodules with an initially nondiagnostic biopsy were eventually determined to be malignant. Table 7 summarizes the subsequent diagnostic evaluation and results for nodules ultimately found to be malignant with initially nondiagnostic pathology. This study was not powered to draw specific conclusions regarding which nondiagnostic pathology results are more or less likely to be ultimately found malignant.

**Table 5:** Types of Benign Pathology Results

Result	No.	%
Granuloma	10	25
Infectious, bacterial	15	38
Infectious, fungal	5	13
Infectious, mycobacterial	2	5
Organizing pneumonia	6	15
Radiation fibrosis	1	3
Lymphoid interstitial pneumonia	1	3
Total	40	100

**Table 6:** Distribution of Ultimate Diagnoses Based on Initial Nondiagnostic Findings

Nondiagnostic Finding (Initial)	Value	Ultimate Diagnosis	
		Malignant	Benign
Atypical cells	25 (48.1)	11 (55.0)	9 (45.0)
Normal lung/airway	16 (30.8)	6 (30.0)	6 (30.0)
Inflammation (acute and chronic)	7 (13.5)	2 (10.0)	3 (15.0)
Fibrosis	3 (5.8)	1 (5.0)	1 (5.0)
Necrosis	1 (1.9)	0 (0.0)	1 (5.0)
Total	52	20	20

Data are presented as No. or No. (%).

**Table 7:** Cancer Types, Diagnostic Methods, and Timing After Initial Nondiagnostic Findings (n = 20)

Types of Initial Nondiagnostic Findings	Ultimate Cancer Type	Diagnostic Test Type	Approximate Time After Initial Bronchoscopy
Atypical cells	Adenocarcinoma	EBUS/ENB	6 mo
Atypical cells	Empirical SBRT	NA	NA
Atypical cells	Squamous cell carcinoma	EBUS/ENB	2 mo
Atypical cells	Adenocarcinoma	Lobectomy	3 wk
Atypical cells	Undifferentiated carcinoma	Lobectomy	1 mo
Atypical cells	Empirical SBRT	NA	NA
Atypical cells	Adenocarcinoma	CT scan-guided biopsy	2 wk
Atypical cells	Carcinoid tumor	Lobectomy	3 wk
Atypical cells	Solitary fibrous tumor	EBUS/ENB	3 wk
Atypical cells	Small cell carcinoma	CT scan-guided biopsy	3 wk
Atypical cells	Adenocarcinoma	Wedge resection	24 mo
Fibrosis	Adenocarcinoma	Wedge resection	1 mo
Inflammation	Adenocarcinoma	EBUS/ENB	24 mo
Inflammation	Adenocarcinoma	CT scan-guided biopsy	2 wk
Normal lung/airway	Adenocarcinoma	Wedge resection	2 mo
Normal lung/airway	Carcinoid tumor	EBUS/ENB	24 mo
Normal lung/airway	Mucinous adenocarcinoma	EBUS/ENB	8 mo
Normal lung/airway	Mucinous adenocarcinoma	EBUS/ENB	5 mo
Normal lung/airway	Adenocarcinoma	EBUS/ENB	7 mo
Normal lung/airway	Squamous cell carcinoma	EBUS/ENB	24 mo

EBUS = endobronchial ultrasound; ENB = electromagnetic navigation bronchoscopy; NA = not applicable; SBRT = stereotactic body radiation therapy.

Table 8 shows sensitivity and specificity diagrams, and values for sensitivity, NPV, and diagnostic accuracy where all lost to follow-up patients are included in the denominator, as was done in this paper. Hypothetical scenarios are also shown where all patients lost to follow-up or dead were false negatives (worst case scenario), all patients lost to follow-up or dead were true negatives (best case scenario), andthe lost to follow-up patients were excluded from the denominator. For illustration, using these best case and worse case scenarios, NPV may be as low as 51.1% or as high as 78.3%, sensitivity may be as low as 75%, and diagnostic accuracy may be as low as 71.4% and as high as 77.1%. Formulas for NPV, positive predictive value, sensitivity, and specificity are shown in Table 9, and the formula for diagnostic accuracy is shown in Table 10.

**Table 8:** Sensitivity, NPV, and Diagnostic Accuracy Under Various Hypothetical Scenarios for Lost to Follow-Up and Patients Who Died

Included in Denominator (N = 227)					
		Malignancy			
		Present	Absent	Sensitivity	87.10
Biopsy result	+	135	0	NPV	70.15
	–	20	47	Diagnostic accuracy	71.37

All Malignant (N = 227)					
		Malignancy			
		Present	Absent	Sensitivity	75.00
Biopsy result	+	135	0	NPV	51.09
	–	45	47	Diagnostic accuracy	71.37

All Benign (N = 227)					
		Malignancy			
		Present	Absent	Sensitivity	87.10
Biopsy result	+	135	0	NPV	78.26
	–	20	72	Diagnostic accuracy	77.09

Totally Excluded (n = 202)					
		Malignancy			
		Present	Absent	Sensitivity	87.10
Biopsy result	+	135	0	NPV	70.15
	–	20	47	Diagnostic accuracy	80.20

NPV = negative predictive value.

## Discussion

In this single-center, medium- to large-sized community hospital study evaluating the performance of DT-ENB for lung nodule biopsy in 227 patients, diagnostic yield using the strict definition was 77.1%. This is consistent with the performance reported in prior studies of DT-ENB. Sensitivity for malignancy was 87.1%, with an NPV for malignancy of 70.1% in the 202 patients who completed 2 years of follow-up CT scan and evaluation.

There were 20 false negatives eventually diagnosed with malignancy from an initially nondiagnostic biopsy, which is exactly one-half of all nondiagnostic biopsies that made it to 2 years of follow-up. Of these patients, 11 had atypical cells on initial biopsy; however, other nonspecific findings were seen including normal lung tissue without discrete pathologic abnormality. Although this study is not powered to detect statistical differences, grossly equivalent number of malignant and benign diagnoses were seen after initial results of atypical cells, fibrosis, acute/chronic inflammation, and normal lung/airway. This highlights the importance of continued follow-up of patients, particularly with nondiagnostic biopsies.

Table 7 briefly describes what clinical and diagnostic follow-up was completed along with the type of malignancy ultimately diagnosed. Two patients underwent empirical stereotactic body radiation therapy given high clinical suspicion for malignancy and atypical cells on pathology after the index procedure and tumor board evaluation. Three patients underwent lobectomy, and 2 patients underwent wedge resection after the index procedure and tumor board evaluation. Three patients underwent CT scan-guided biopsy because of high clinical suspicion, which confirmed malignancy. One patient underwent a wedge resection after nodule growth after a prolonged period of stability on CT scan, confirming a carcinoid tumor. The remaining 9 patients underwent a repeat EBUS/ENB in the interval because of growth on CT monitoring, which was diagnostic. This descriptive information highlights the importance of an organized lung nodule program and the benefits of a multidisciplinary tumor board.

We think the diagnostic yield and performance characteristics reported in this study are applicable to the average pulmonary practice for multiple reasons. The operators were all attending pulmonologists with a variable number of ENBs performed in their careers. Furthermore, as a community hospital, the prevalence of cancer in this population may be lower than in some academic cancer centers. Additionally, the 2018 to 2022 age-adjusted incidence rate of lung and bronchus cancer in the area was 60.9 per 100,000 population, consistent with the national incidence rate of 53.3 per 100,000 population.^26^ At our community hospital, the workflow for DT-ENB has been efficient, and in our opinion, feasible to implement at other community hospitals.

The biggest strengths of this study are the inclusion of 2 years of follow-up data on 202 patients and strict/conservative definitions of diagnostic yield and diagnostic accuracy. To our knowledge, this is the longest available follow-up data available using this platform for DT-ENB. Our strict definition of what constitutes a benign finding and designation of nonspecific results such as atypical cells as nondiagnostic meets criteria recommended for advanced diagnostic bronchoscopy outcomes by the ATS and CHEST in 2023.^8^ Despite using the strict definition for diagnostic yield, our yield of 77.1% was higher than reported in the NAVIGATE study (67.8%).^5^ Our calculated diagnostic accuracy of 71.4% was consistent with the VERITAS trial (79%), but is additionally reinforced by 2 years of follow-up.^7^

One study limitation is the single-center, retrospective design. Variation in operator yield was not assessed because most procedures (156 procedures) were performed by the study’s most experienced operator, with the rest being performed by the remaining 3 operators (49, 13, and 9 procedures). Thus, these sample sizes were not large enough to draw accurate conclusions on interoperator variability. In addition, results may not be entirely applicable to less experienced operators, or for example, centers where clinical fellows are performing the procedure. This study is also underpowered to analyze patient outcomes after specific nondiagnostic biopsy results (eg, atypical cells). More data are needed on the significance and outcomes of these findings. Approximately 11% of patients were excluded from the final 2-year sensitivity and NPV analysis: 13 patients were lost to follow-up, and 12 patients died before a conclusive diagnosis. It is unclear how many of these patients would have been diagnosed with malignancy with ongoing clinical and radiographic monitoring and evaluation; therefore, the reported sensitivity, NPV, and diagnostic accuracy may be overestimated or underestimated. For example, if all patients who died or were lost to follow-up were found to have malignancy (all false negatives), the diagnostic accuracy would be unaffected, but the NPV and sensitivity would be lower at 51.1% and 75%, respectively. In the scenario that all patients had confirmed benign nodules, the sensitivity would be unaffected, but calculated diagnostic accuracy and NPV would be higher at 77.1% and 78.3%, respectively. As this illustrates, our value for diagnostic accuracy is likely an underestimate.

## Interpretation

In this community hospital-based retrospective observational performance evaluation of DT-ENB, the diagnostic yield was 77.1%, the 2-year diagnostic accuracy was 71.4%, and the 2-year sensitivity and NPV for malignancy were 87.1% and 70.1%, respectively. A strict definition of diagnostic yield and a conservative definition of diagnostic accuracy were used. A higher initial diagnostic yield was seen for larger nodules, with a median nodule size for nondiagnostic procedures of 1.5 cm (95% CI, 1.25-2.15 cm) and a median nodule size for diagnostic procedures of 2.2 cm (95% CI, 1.4-4.0 cm). In addition to presenting performance with 2 years of follow-up data, it demonstrates the importance of an organized institutional lung nodule program.

## Funding/Support

The authors have reported to *CHEST Pulmonary* that no funding was received for this study.

## Financial/Nonfinancial Disclosures

None declared.
